# Simplified Analysis of Native Steroid Esters in Dried Blood Spots by LC–MS^3^

**DOI:** 10.1002/jms.5188

**Published:** 2025-10-08

**Authors:** Andreas Thomas, Jasmin Thelen, Panagiotis Sakellariou, Mario Thevis

**Affiliations:** ^1^ Institute of Biochemistry/Center for Preventive Doping Research German Sport University Cologne Cologne Germany; ^2^ European Monitoring Center for Emerging Doping Agents (EuMoCEDA) Cologne/Bonn Germany

**Keywords:** doping control, dried blood spots, LC–MS/MS, LC–MS^3^, testosterone

## Abstract

Steroidal esters (e.g., testosterone, nandrolone and boldenone esters) belong to the substance class prohibited in professional sport that is particularly frequently abused due to its considerable performance‐enhancing effects. Thus, a high number of adverse analytical findings in doping controls is reported every year. Unfortunately, the analysis of steroids that are not exclusively of exogenous nature and, in particular, their differentiation from endogenously produced steroids is very time‐consuming and resource‐intensive (necessitating isotope‐ratio mass spectrometric analysis). The direct detection of the applied (unequivocally exogenous) steroidal esters in blood or dried blood spots (DBS) can offer a much simpler approach. In the present project, the analysis of 17 underivatized steroidal esters (composed of testosterone, nandrolone and boldenone species) is reported, employing liquid chromatography‐(low‐resolution)‐MS^n^. The results show that all included esters can be selectively and sensitively detected in MS^3^ mode at sub‐ng/mL levels in DBS. The detection limits for most analytes extracted from a single spot were below 0.1 ng/mL, and recovery rates were determined at 40%–80%. The overall procedure was controlled using four different stable isotope‐labelled internal standards. Notably, the obtained results are largely independent from the sampling device, and the method works for cellulose‐based DMPK cards as well as polymer‐based TASSO devices. Proof‐of‐concept and applicability of the method to authentic samples were demonstrated by analysing post‐administration samples collected after an oral administration of 80 mg of testosterone undecanoate.

## 1 Introduction

The detection of anabolic androgenic steroids (AAS) in sports drug testing remains an important and critical component of anti‐doping efforts worldwide. Traditionally, urine has served as the primary biological matrix for the detection of steroid abuse; however, alternative matrices such as dried blood spots (DBS) are gaining traction due to their minimally invasive sampling, ease of transport and potential for improved analyte stability [1]. Steroid esters, commonly administered via intramuscular injection or oral administration (e.g., testosterone undecanoate), are among the most misused AAS [2]. These esters are lipophilic derivatives of anabolic steroids, typically esterified at the 17β‐hydroxyl group (e.g., testosterone, nandrolone or boldenone). The esterification prolongs the steroids' half‐life by increasing their lipophilic properties and, thus, allowing for sustained release from muscle depots after injection. In blood, steroid esters are generally susceptible to enzymatic hydrolysis by esterases, converting them into their active free steroid forms [2, 3, 4]. This hydrolysis can occur rapidly in whole blood, particularly when stored at room temperature, more slowly under cold storage at 4°C or −20°C, respectively, and this hydrolysis is significantly suppressed in DBS due to reduced enzymatic activity in dry conditions [4].

While these nonnatural esters are commonly not observed as intact analytes in urine, the analysis for those compounds in urinary specimens focuses on the deesterified steroids, followed by dedicated analyses that allow for differentiating these from their endogenous counterparts (where necessary). These follow‐up analyses for endogenous AAS mainly rely on isotope‐ratio‐mass spectrometry (IRMS) [5]. The direct measurement of the nonnatural ester form in DBS represents a much easier and faster approach, [2, 3] with the limitation that the detection window for steroidal esters in DBS has been found to be substantially shorter than urine analyses employing IRMS [6]. Nevertheless, several assays to detect steroid esters in DBS by means of liquid chromatography coupled to mass spectrometry (LC–MS/MS) have been developed recently [3, 4, 6, 7, 8, 9, 10, 11, 12]. The main challenges arise from the complex matrix in combination with the required sensitivity due to the very low levels of steroidal esters in blood. Recent application studies with different steroid esters have demonstrated that the whole blood concentrations usually fall within the low nanogram per milliliter range, and most analytical methods have accordingly been designed to target these levels [10, 13]. To reach these requirements, most assays use derivatisation agents to enhance the ionisation efficiency of the target analytes in electrospray ionisation (ESI) in combination with sophisticated chromatographic conditions [5, 9, 10, 11, 12, 13]. Notably, also automated extraction of DBS (cellulose cards only) with subsequent analysis of native steroid esters was published recently [8].

The introduction of new advancements in mass spectrometry, particularly high‐resolution and tandem mass spectrometry (HRMS and MS/MS), has enabled the sensitive and specific detection of these esters even at trace levels. When coupled with micro‐sampling techniques like DBS, mass spectrometry offers a promising approach for out‐of‐competition and in‐competition testing, especially where traditional sample collection is impractical.

This study focuses on the development and validation of a robust analytical method for the detection of native steroid esters from DBS using LC–MS^3^. Emphasis was placed on method sensitivity, selectivity and its application to realistic doping control scenarios.

## 2 Experimental

### 2.1 Chemicals and Reagents

Methanol and acetonitrile in LC–MS grade were purchased from Sigma (Schnelldorf, Germany). The TASSO‐M20 polymer sampling devices were obtained from TASSO (Seattle, USA), and the cellulose‐based DMPK‐C cards were from Qiagen (Hilden, Germany). Water in ultrapure quality was used for preparing aqueous solutions.

The reference substances for testosterone, testosterone acetate, testosterone propionate, testosterone isocaproate, testosterone caproate, testosterone enantate, testosterone cypionate, testosterone phenylpropionate, testosterone decanoate, testosterone undecanoate, nandrolone, nandrolone decanoate, nandrolone benzoate, boldenone, boldenone propionate, boldenone cypionate, boldenone undecanoate, ^2^H_3_‐testosterone, ^2^H_3_‐testosterone enantate, ^2^H_3_‐nandrolone caproate and ^2^H_3_‐testosterone undecanoate were purchased from Sigma (Schnelldorf, Germany), LGC Promochem (Tokyo, Japan), Cayman Chemicals (Wesel, Germany) and Biosynth (Berlin, Germany).

### 2.2 Samples

The samples used for the validation experiments were obtained from healthy male and female volunteers who had not taken any medication during the previous 48 h. Written informed consent was obtained from all volunteers, and the study was approved by the local ethics committee (DSHS No.: 139/2021). Further, one healthy volunteer (male, 51 years, 78 kg) orally administered 2 capsules of Andriol (each containing 40 mg of testosterone undecanoate). Samples were collected using DMPK cards (20 μL) before and at 2, 4, 6, 8, 12 and 22 h after administration. The administration samples are from an earlier study (ref no 1217) [4].

### 2.3 Sample Preparation

The sample preparation procedure was essentially adapted from an earlier study [7]. Briefly, the dried blood samples (TASSO polymer or 8 mm punch from the cellulose‐based card) were fortified with 5 μL of internal standard (ISTD) solution (containing 2 ng/mL ^2^H_3_‐testosterone, 8 ng/mL ^2^H_3_‐nandrolone caproate, 16 ng/mL ^2^H_3_‐testosterone enanthate and 16 ng/mL ^2^H_3_‐testosterone undecanoate) in an Eppendorf tube and subsequently extracted with 400 μL of a mixture of methanol (80%)/water (20%) in an ultrasonic bath for 20 min. The extraction solution was transferred to a new tube and evaporated in a vacuum centrifuge at approximately 40°C to dryness. After reconstitution in 80 μL of a mixture of methanol (60%)/water (40%), the samples were vortexed and centrifuged for 5 min at 13 000 rpm. The supernatant was transferred to an HPLC vial for injection.

### 2.4 Validation

The method was validated according to the requirements of current World Anti‐Doping Agency (WADA) documents, including the parameters selectivity, limit of detection (LOD), reliability, recovery, stability, carryover and robustness [14]. For selectivity, 10 samples from healthy volunteers without any medical treatment were analysed (10 TASSO samples and 10 DMPK card samples) and monitored for interfering signals (aimed detection rate 0/10 or 0%). For reliability, another 10 samples from different volunteers were fortified with all target analytes at 1 ng/mL and analysed (TASSO and cards, aimed detection rate 10/10 or 100%). This 1 ng/mL concentration was set as a provisional 100% level (comparable to a minimum required performance level, MRPL). The LOD for TASSO was estimated via the detection rate of six samples from different volunteers fortified with all target analytes at 0.5, 0.25, 0.1 and 0.05 ng/mL (corresponding to 50%, 25%, 10% and 5% MRPL, aimed detection rate 6/6 or 100%). For cellulose cards, six samples were analysed and the concentration levels were set to 0.5 and 0.1 ng/mL (aimed detection rate 6/6 or 100%). To investigate the recovery, six samples (TASSO) were fortified before extraction and six samples after the extraction at a concentration of 2 ng/mL. Thus, recovery was computed as the loss of target analyte during the sample preparation procedure in percent. The stability in the autosampler was tested after storage in the vial at 10°C for 72 h. Carryover in the LC–MS system from high concentrated samples (4 ng/mL) to the subsequent blank samples was tested in three repetitions.

### 2.5 Liquid Chromatography/Mass Spectrometry (LC–MS)

LC–MS was performed on a Vanquish UHPLC liquid chromatograph coupled to a Thermo Stellar mass spectrometer (both Thermo Fisher Bremen, Germany). Solvent A was 0.1% formic acid in water, and 0.1% formic acid in methanol was used as solvent B. The gradient started at 40% of B, increased to 99% of B in 10 min, ran isocratic at 99% of B for 2.5 min, with subsequent re‐equilibration at 40% of B for 2.5 min. Thus, the overall runtime was 15 min, with a flow rate set to 350 μL/min, using an Accucore C18, 100 × 2.1 mm with 2.6 μm particle size analytical column (Thermo Fisher Bremen, Germany). A volume of 10 μL of the sample was used for injection.

The mass spectrometer was equipped with a HESI auto ready ion source supported with nitrogen as spray gas. The temperatures were set to 550°C for the vaporizer and at 325°C for the ion transfer tube. Positive capillary voltage was set to 3.5 kV. The gas supply for the collision cells was nitrogen and helium of 5.0 grade. The instrument was used in tMS^n^ mode with a quadrupole isolation window of 1.5 Da, and both activation types (higher energy collision‐induced dissociation: HCD, and collision‐induced dissociation: CID) were enabled (see Table 1). The precursors were first activated by CID, and the resulting product ions were further processed with HCD. For precursor ions, the CID activation time was set to 2 ms and the activation Q at 0.25. HCD and CID collision energies were set as shown in Table 1. For the dissociation of the product ion, the CID activation time was set to 10 ms with an activation Q of 0.25. For all experiments, the expected retention times of the respective esters were scheduled applying a time window of 2 min. Exemptions were made for testosterone and the respective ISTD (^2^H_3_‐testosterone), which were analysed in MS^2^ mode.

**TABLE 1:** Main mass spectrometric and chromatographic parameters.

Group	Analyte	Precursor ion [*m/z*]	CID [%]	Product ion MS^2^ [*m/z*]	HCD [%]	Product ion MS^3^ [*m/z*]	HCD [%]	~ Retention time [min]
Steroid esters	Testosterone	289.2	38	109	—	—	—	5.21
	Testosterone acetate	317.3	28	253	48	197	48	7.44
	Testosterone propionate	345.3	28	253	48	197	48	8.24
	Testosterone isocaproate	387.3	28	253	48	197	48	9.73
	Testosterone caproate	387.3	28	253	48	197	48	9.84
	Testosterone enantate	401.3	28	253	48	197	48	10.26
	Testosterone cypionate	413.3	28	253	48	197	48	10.39
	Testosterone phenylpropionate	421.3	28	253	48	197	48	9.45
	Testosterone decanoate	443.4	28	253	48	197	48	11.26
	Testosterone undecanoate	457.4	28	253	48	197	48	11.55
	Nandrolone	275.2	28	239	48	197	48	4.69
	Nandrolone decanoate	429.4	28	239	48	128	48	11.08
	Nandrolone benzoate	379.3	28	239	48	197	48	9.01
	Boldenone	287.2	28	269	45	121	45	4.49
	Boldenone propionate	343.3	28	269	45	173	45	7.52
	Boldenone cypionate	411.3	28	269	45	121	45	9.86
	Boldenone undecanoate	455.4	28	269	45	121	45	11.16
ISTD	^*2*^ *H*_*3*_ *‐Testosterone*	*292.2*	*38*	*109*	*—*	*—*	*—*	*5.19*
	^*2*^ *H*_*3*_ *‐Testosterone enantate*	*404.3*	*28*	*256*	*48*	*213*	*48*	*10.25*
	^*2*^ *H*_*3*_ *‐Nandrolone caproate*	*376.3*	*28*	*241*	*48*	*197*	*48*	*9.54*
	^*2*^ *H*_*3*_ *‐Testosterone undecanoate*	*460.4*	*28*	*256*	*48*	*—*	*48*	*11.54*

## 3 Results and Discussion

The here presented assay employs MS^3^ experiments coupled to (low‐resolution) mass spectrometry for sensitive detection of steroid esters from DBS for doping controls without derivatisation. In comparison to existing assays (mainly employing derivatisation steps and/or high resolution mass spectrometry), the sensitivity in this assay is competitive or even better [7, 11, 12, 13]. Noteworthy, the required specificity under the chosen conditions was reached in MS^3^ mode for nearly all of the target esters only. The analysis in MS^2^ mode does not enable the effective measurement in the sub nanogram per milliliter range due to lack of specificity. A comparative measurement of a fortified QC sample (at 0.1 ng/mL) in MS^3^‐resp. MS^2^‐mode is shown in Figure S1. Here, the gain in specificity is demonstrated for the analysis of testosterone propionate, yielding significantly better signal‐to‐noise for the selected product ions in MS^3^‐mode. Another benefit of the native analysis (without derivatisation) is the potential to expand the assay to several other classes of doping substances [7].

### 3.1 Validation

**FIGURE 1:**
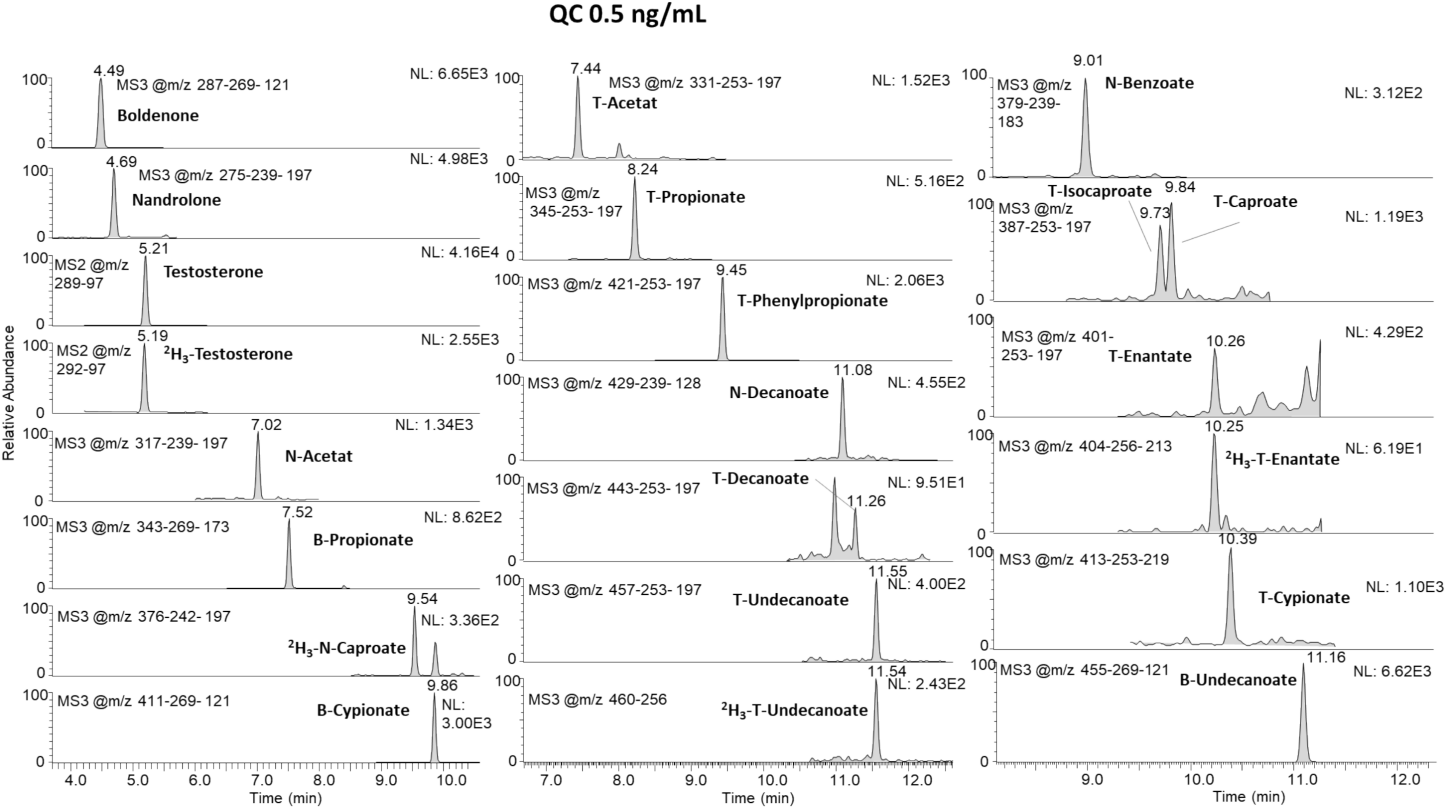
Extracted MS^3^ chromatograms of a fortified DBS sample (quality control at 0.5 ng/mL). T = testosterone; N = nandrolone; B = boldenone.

**FIGURE 2:**
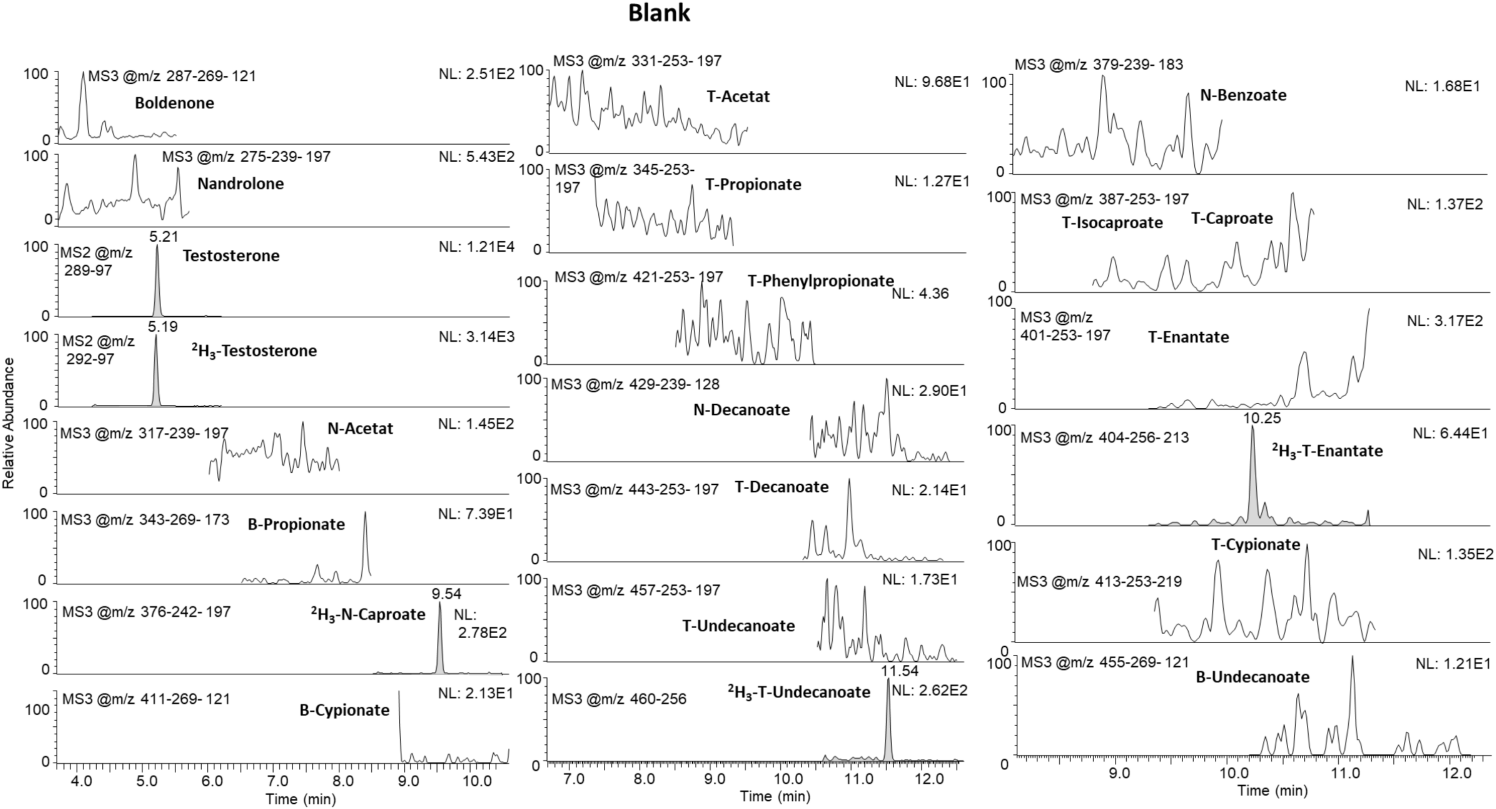
Extracted MS^3^ chromatograms of a blank DBS sample fortified with ISTDs only. T = testosterone; N = nandrolone; B = boldenone.

Main results of the validation are shown in Tables 2a and 2b for TASSO sampling devices and cellulose‐based cards, respectively. The extracted ion chromatograms of the blank samples from healthy male and female volunteers showed no interfering signals for all target analytes at the respective retention times, and all signals for the ISTDs were detected; thus, selectivity for TASSO and cellulose‐based cards was proven. Endogenous testosterone was detected in all samples (males and females). At the proposed MRPL of 1 ng/mL, all target steroid esters were detected in all 10 TASSO and 10 card samples (detection rate: 100%). Figures 1 and 2 illustrate representative chromatograms of a blank sample and a sample fortified at 0.5 ng/mL with all target analytes. Detailed detection rates are shown in Table 2a and 2b, indicating LODs at 0.1 ng/mL or below for most of the included steroids. Recoveries ranged from 43% to 85% for the different esters, with an apparent lower recovery rate for longer‐chain steroid esters. This observation is attributable to the reduced solubility of these nonpolar substances; however, the accomplished detection limits proved to be fit‐for‐purpose for sensitively analysing all included steroid esters. Carryover from highly concentrated samples (4 ng/mL) to the subsequent injection was below 1%, and the extracts were found to be stable for at least 3 days at 10°C in the autosampler (detection rate: 100%).

**TABLE 2a:** Validation results for DBS TASSO devices.

Group	Analyte	100% QC TASSO		50% QC TASSO		25% QC TASSO		10% QC TASSO		5% QC TASSO		Selectivity detection rate TASSO *n* = 10	Recovery	Carry over TASSO	Extract stability TASSO (3 days @10°C)
		C [ng/mL]	Detection rate [%]	C [ng/mL]	Detection rate [%]	C [ng/mL]	Detection rate [%]	C [ng/mL]	Detection rate [%]	C [ng/mL]	Detection rate [%]		TASSO *n* = 6 + 6		
Steroid esters	Testosterone acetate	1	100	0.5	100	0.25	100	0.1	100	0.05	100	0/10	77%	< 1%	100
	Testosterone propionate	1	100	0.5	100	0.25	100	0.1	100	0.05	100	0/10	67%	< 1%	100
	Testosterone isocaproate	1	100	0.5	100	0.25	100	0.1	100	0.05	100	0/10	73%	< 1%	100
	Testosterone caproate	1	100	0.5	100	0.25	100	0.1	100	0.05	100	0/10	77%	< 1%	100
	Testosterone enantate	1	100	0.5	100	0.25	100	0.1	100	0.05	100	0/10	84%	< 1%	100
	Testosterone cypionate	1	100	0.5	100	0.25	100	0.1	100	0.05	100	0/10	72%	< 1%	100
	Testosterone phenylpropionate	1	100	0.5	100	0.25	100	0.1	100	0.05	100	0/10	72%	< 1%	100
	Testosterone decanoate	1	100	0.5	100	0.25	100	0.1	33	0.05	0	0/10	52%	< 1%	100
	Testosterone undecanoate	1	100	0.5	100	0.25	100	0.1	100	0.05	100	0/10	43%	< 1%	100
	Nandrolone	1	100	0.5	100	0.25	100	0.1	101	0.05	100	0/10	74%	< 1%	100
	Nandrolone decanoate	1	100	0.5	100	0.25	100	0.1	100	0.05	100	0/10	54%	< 1%	100
	Nandrolone benzoate	1	100	0.5	100	0.25	100	0.1	100	0.05	100	0/10	73%	< 1%	100
	Boldenone	1	100	0.5	100	0.25	100	0.1	101	0.05	100	0/10	85%	< 1%	100
	Boldenone propionate	1	100	0.5	100	0.25	100	0.1	100	0.05	100	0/10	77%	< 1%	100
	Boldenone cypionate	1	100	0.5	100	0.25	100	0.1	100	0.05	100	0/10	74%	< 1%	100
	Boldenone undecanoate	1	100	0.5	100	0.25	100	0.1	100	0.05	100	0/10	54%	< 1%	100

**TABLE 2b:** Validation results for DBS cellulose cards (DMPK‐C).

Group	Analyte	100% QC Card		50% QC Card		10% QC Card		Selectivity detection rate cardn = 10
		C [ng/mL]	Detection rate [%]	C [ng/mL]	Detection rate [%]	C [ng/mL]	Detection rate [%]	
**Steroid esters**	Testosterone acetate	1	100	0.5	100	0.1	100	0/10
	Testosterone propionate	1	100	0.5	100	0.1	100	0/10
	Testosterone isocaproate	1	100	0.5	100	0.1	100	0/10
	Testosterone caproate	1	100	0.5	100	0.1	100	0/10
	Testosterone enantate	1	100	0.5	100	0.1	100	0/10
	Testosterone cypionate	1	100	0.5	100	0.1	100	0/10
	Testosterone phenylpropionate	1	100	0.5	100	0.1	100	0/10
	Testosterone decanoate	1	100	0.5	100	0.1	33	0/10
	Testosterone undecanoate	1	100	0.5	100	0.1	100	0/10
	Nandrolone	1	100	0.5	100	0.1	101	0/10
	Nandrolone decanoate	1	100	0.5	100	0.1	100	0/10
	Nandrolone benzoate	1	100	0.5	100	0.1	100	0/10
	Boldenone	1	100	0.5	100	0.1	101	0/10
	Boldenone propionate	1	100	0.5	100	0.1	100	0/10
	Boldenone cypionate	1	100	0.5	100	0.1	100	0/10
	Boldenone undecanoate	1	100	0.5	100	0.1	100	0/10

### 3.2 Post‐Administration Samples

**FIGURE 3:**
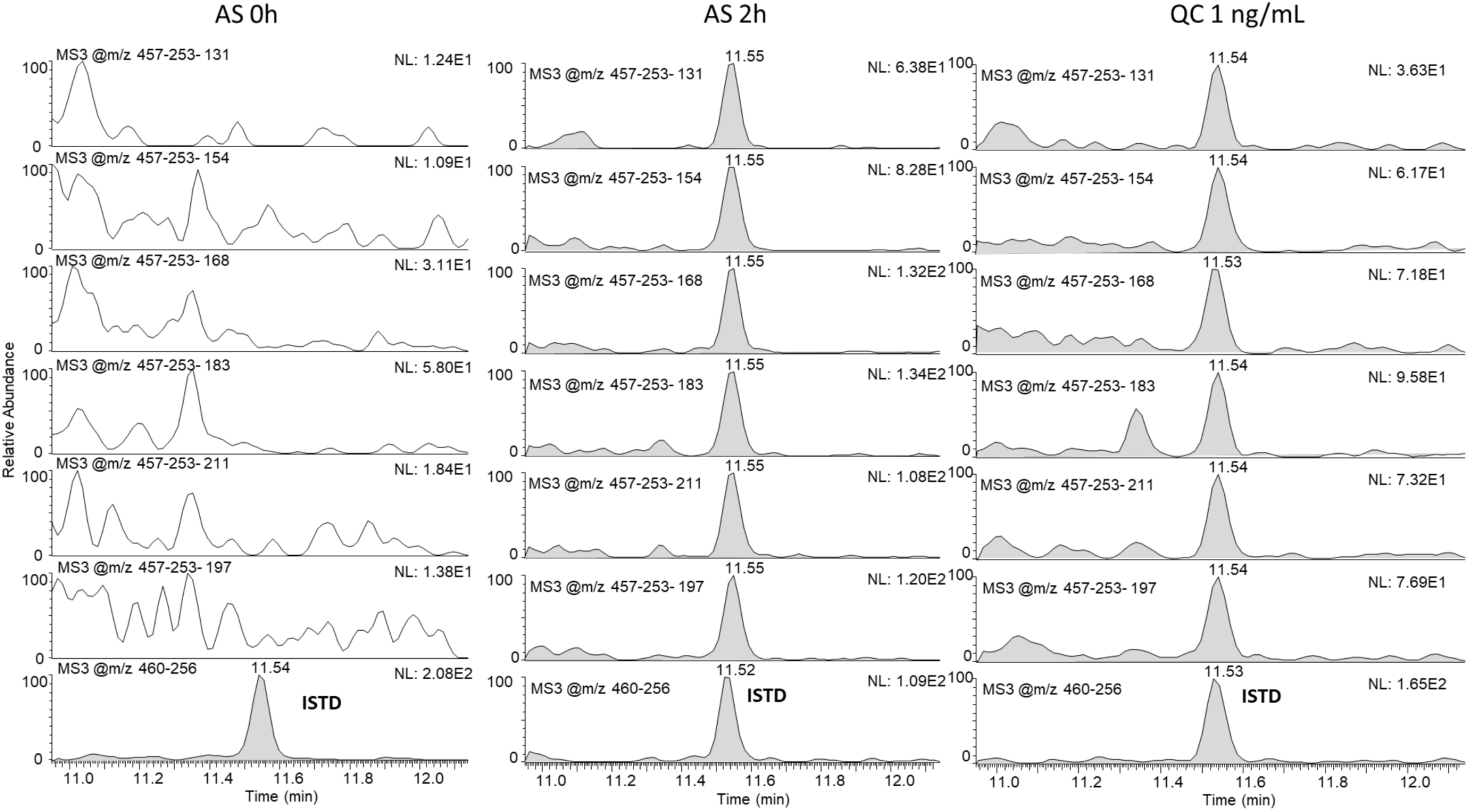
Extracted MS^3^ chromatograms for testosterone undecanoate of a blank DBS sample (AS 0 h), a post‐administration sample (AS 2 h), and a quality control sample (QC, fortified at 1 ng/mL).

The analysis of post‐administration samples after oral application of 80 mg of Andriol provided authentic test results beyond those obtained from spiked test method validation samples. The respective extracted ion chromatograms are shown in Figure 3. Here, six diagnostic ion traces from different MS^3^‐derived product ions are shown, which impressively enable the detection (and confirmation) of testosterone undecanoate in the sample collected 2 h after administration. Notably, the data shown are extracted from the original initial testing procedure as described above (not a targeted confirmation method), including all target esters. The analysis of the samples after oral administration of testosterone undecanoate showed rather short detection windows; after 6 to 8 h post‐administration, the drug was no longer detectable in DBS (< 50 pg./mL). This is in accordance with earlier studies, and other routes of application (e.g., intramuscular injection) will certainly yield much longer detection windows.

### 3.3 Doping Control Aspects

**FIGURE 4:**
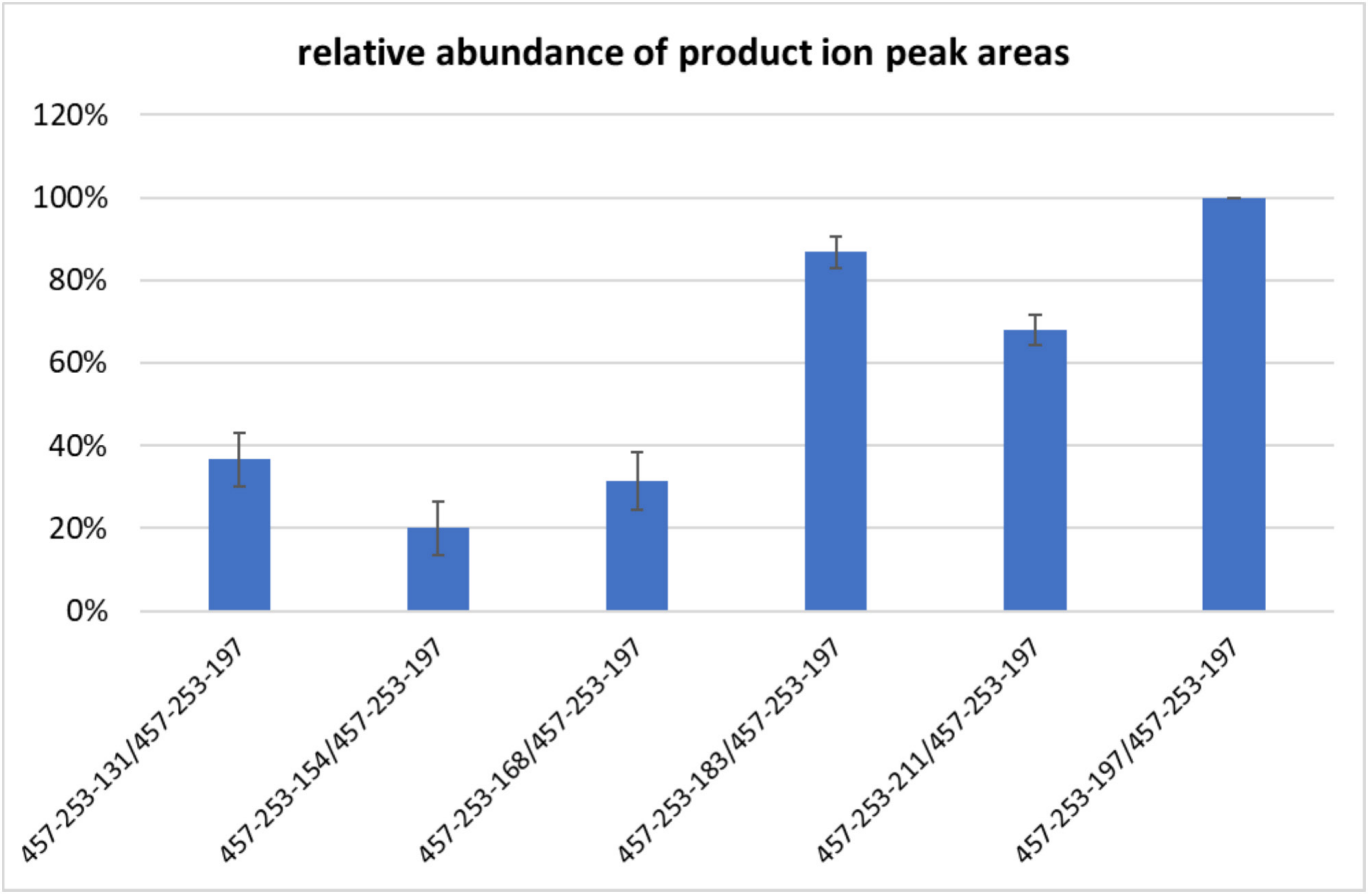
Mean product ion peak area ratios in MS^3^ (data acquired over the working range of the method from 0.1 to 1 ng/mL) computed from the precursor ion at *m/z* 457 and the first product ion at *m/z* 253 for testosterone undecanoate (see also Figure 3). The black bars indicate standard deviation for the peak areas for the respective product ion pair.

As already mentioned before, the detection of steroid esters in blood samples represents a direct adverse analytical finding (AAF) and, accordingly, an anti‐doping rule violation. In case of a suspicious result in the initial testing, WADA requirements mandate a subsequent confirmatory analysis dedicated to the individual steroid ester from another spot [14, 15]. WADA technical documents provide detailed criteria for the liquid chromatographic as well as the mass spectrometric parameters, which must be fulfilled before the final identification of a prohibited substance in a doping control sample can be confirmed [15]. Besides the evaluation of the (relative) retention times of the analyte peak in the sample and the quality control sample (fortified with the respective target analyte), several distinct mass spectrometric criteria are also specified. One such criterion is the acceptance range for the product ion peak area ratios of at least two diagnostic product ions, and thus, stable and reproducible conditions in the collision cell are crucial. The herein employed mass spectrometer composed of hyphenated quadrupole, multipole and dual‐pressure linear ion trap units allows for combined activation types (CID and HCD), for which such stability data have been scarce [16]. To obtain at least presumptive information about the stability of the peak area of product ion ratios relevant to this project, the standard deviations for six product ion ratios (exemplarily for testosterone undecanoate, see Figure 3) were computed for all samples of the LOI measurements (at 1.0, 0.5, 0.25 and 0.1 ng/mL, *n* = 28). Figure 4 shows the obtained data with the most abundant signal at *m/z* 457‐253‐197 set as 100%. The calculated standard deviations are shown as black bars and all range below 15%, with no significant bias due to the different concentrations (in the working range from 0.1 to 1 ng/mL). These data strongly suggest that the approach will provide stable peak area ratios of product ions as required for confirmatory analyses, especially considering that the obtained data in Figure 4 were recorded from the analysis of the validation samples using the described multi‐target method (> 20 esters including ISTDs). In case of applying targeted confirmation methods dedicated to a single steroid ester only, the increased number of acquired data points over the peak is expected to further improve the robustness of the determined peak area ratios.

## 4 Conclusion

DBS sampling offers an interesting new tool in sports drug testing due to several significant benefits in the doping control process (minimally invasive, easy to transport, high analyte stability, etc.). However, from the laboratory's perspective, new challenges arise with respect to the very low sample volume (< 20 μL, 3–4 spots) and the complex matrix (whole blood). Although enormous progress in the analysis of DBS has already been made in recent years, there is still a need for more sensitivity and specificity. The present approach demonstrates how these requirements can be met by applying a hybrid quadrupole/multipole/ion trap‐based mass spectrometer in MS^3^ mode for the analysis of prohibited steroid esters. The method presented here for native steroids yields comparable or even better results compared to earlier assays, without using analyte derivatisation [7]. This enables analysing the same extract for numerous additional prohibited substances from other classes of the Prohibited List, without the need of preparing another DBS, which significantly increases the effectiveness of the approach [7].

## Conflicts of Interest

The authors declare no conflicts of interest.

## Supporting information


**Figure S1:** Comparison of MS^3^‐mode (CID‐HCD) and MS^2^‐mode (CID) for the analysis of a fortified QC sample (at 0.1 ng/mL) with a significantly better signal‐to‐noise for the signals at 8.22 min for testosterone propionate.

## Data Availability

The data that support the findings of this study are available from the corresponding author upon reasonable request.
