# Supplementary material for: Simplified Analysis of Native Steroid Esters in Dried Blood Spots by LC–MS3
Source: J Mass Spectrom. 2025 Oct 8;60(11):e5188. doi: 10.1002/jms.5188 (PMC12507138; doi:10.1002/jms.5188)
Supplement: Supplementary file 1 — Figure S1: Comparison of MS3‐mode (CID‐HCD) and MS2‐mode (CID) for the analysis of a fortified QC sample (at 0.1 ng/mL) with a significantly better signal‐to‐noise for the signals at 8.22 min for testosterone propionate. [file JMS-60-e5188-s001.docx]

Supplemental Information

**Simplified analysis of native steroid esters in dried blood spots by LC-MS^3^**

Andreas Thomas^1^*, Jasmin Thelen^1^, Panagiotis Sakellariou^1^, Mario Thevis^1,2^

^1^Institute of Biochemistry / Center for Preventive Doping Research, German Sport University Cologne, Germany

^2^European Monitoring Center for Emerging Doping Agents (EuMoCEDA), Cologne/Bonn, Germany

*Correspondence

Andreas Thomas, PhD

Institute of Biochemistry / Center for Preventive Doping Research

German Sport University Cologne

Am Sportpark Müngersdorf 6

50933 Cologne

Germany

Tel.: 0221-49827072

Fax: 0221-49827071

a.thomas@biochem.dshs-koeln.de


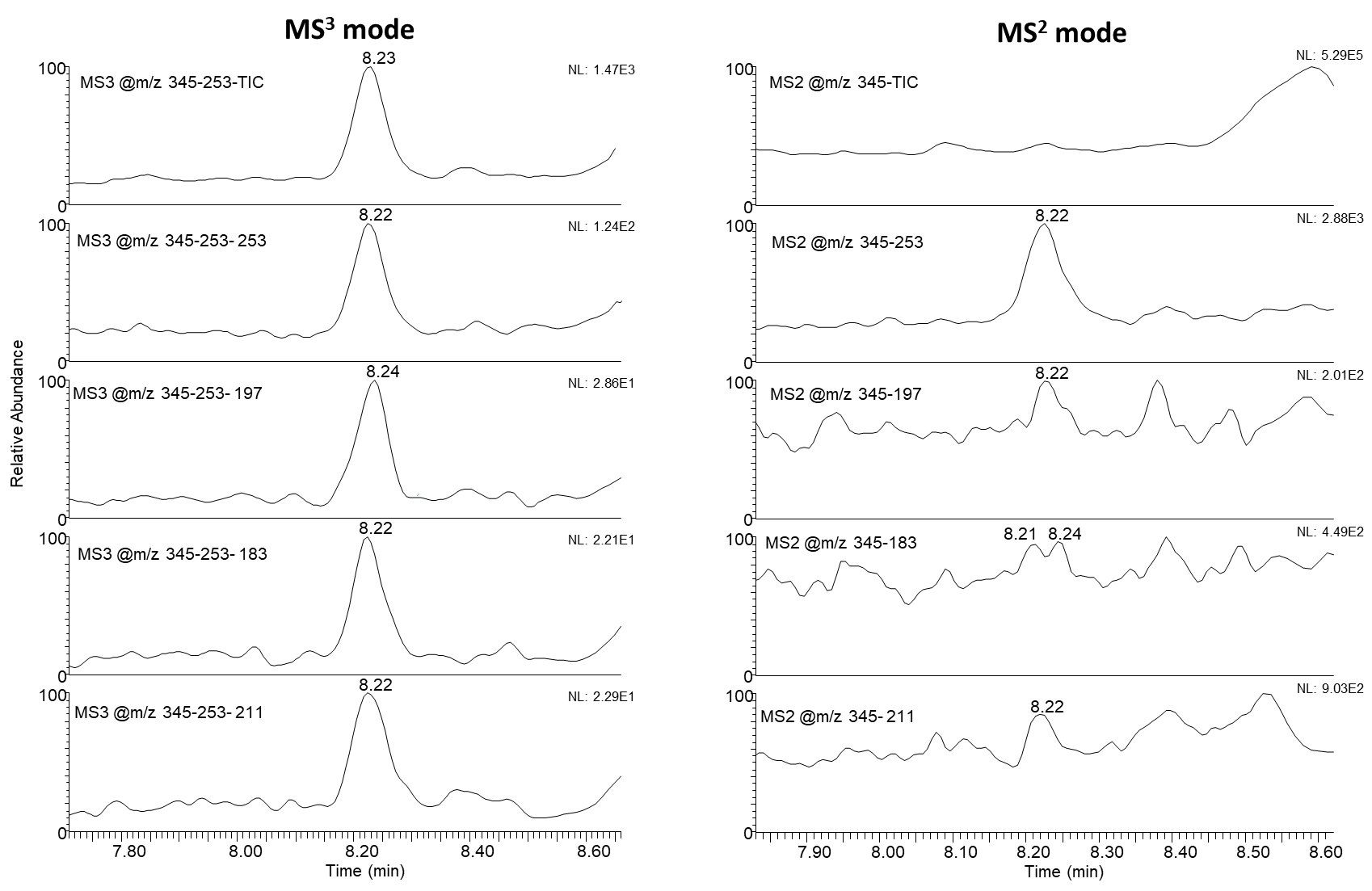


Fig. S1: Comparison of MS^3^-mode (CID-HCD) and MS^2^-mode (CID) for the analysis of a fortified QC sample (at 0.1 ng/mL) with a significantly better signal-to-noise for the signals at 8.22 min for testosterone propionate.
